# Supplementary material for: Empirical methods for controlling false positives and estimating confidence in ChIP-Seq peaks
Source: BMC Bioinformatics. 2008 Dec 5;9:523. doi: 10.1186/1471-2105-9-523 (PMC2628906; doi:10.1186/1471-2105-9-523)
Supplement: Additional File 1 — A variety of html documents from the USeq web site detailing the available applications, their best usage, output file type descriptions, command line menus, etc. [file 1471-2105-9-523-S1.zip › USeqUserGuides/JavaDoc.zip!/JavaDoc/trans/graphics/RankedSetAnalysis.html]

RankedSetAnalysis


|  |  |  |
| --- | --- | --- |
| **Overview** \| **Package** \| **Class** \| **Use** \| **Tree** \| **Deprecated** \| **Index** \| **Help** | |  |
| **PREV CLASS**   **NEXT CLASS** | **FRAMES**    **NO FRAMES**     **All Classes** |  |
| SUMMARY: NESTED \| FIELD \| CONSTR \| METHOD | DETAIL: FIELD \| CONSTR \| METHOD |  |


---


## trans.graphics Class RankedSetAnalysis

```
java.lang.Object
  trans.graphics.RankedSetAnalysis
```

---

``` public class RankedSetAnalysis extends java.lang.Object ```

Performs set analysis on genomic regions with a visual display.

---

**Constructor Summary**

`RankedSetAnalysis(java.lang.String[] args)`


| **Method Summary** | |
| --- | --- |
| `static boolean` | `containsMatchedRankIntersect(GenomicRegion r, int testRank)`             Checks to see if among the hits to a particular GenomicRegion they have an equal or smaller rank. |
| `double` | `fractionTotalIntersection(GenomicRegion[] r)`             Returns the total fraction of intersection. |
| `org.jfree.chart.JFreeChart` | `getChart()` |
| `org.jfree.chart.ChartPanel` | `getChartPanel()` |
| `GenomicRegion[]` | `getRegionsOne()` |
| `GenomicRegion[]` | `getRegionsTwo()` |
| `java.io.File` | `getSetOneFile()` |
| `java.io.File` | `getSetTwoFile()` |
| `TextFrame` | `getTextFrame()` |
| `void` | `intersect()` |
| `boolean` | `intersectRegions()`             Loads regions with references to intersection partners. |
| `boolean` | `isSavePngs()` |
| `static void` | `main(java.lang.String[] args)` |
| `void` | `makeRankedHitsGraph()` |
| `static GenomicRegion[]` | `parseGenomicRegionsFile(java.io.File f)`             Parses a regions file. |
| `static void` | `printDocs()` |
| `void` | `printRankedIntersection(int[] hits)`             Prints to screen the results of rankedIntersection(GenomicRegion[] r). |
| `void` | `printRankedIntersectionAll(GenomicRegion[] r)`             Rank analysis where each regions is check against the entire other list. |
| `void` | `processArgs(java.lang.String[] args)`             This method will process each argument and assign any new varibles |
| `static int[]` | `rankedIntersection(GenomicRegion[] r)`             Calculates the number of hits in the ranked matched list. |
| `void` | `setChartPanel(org.jfree.chart.ChartPanel chartPanel)` |

**Methods inherited from class java.lang.Object**

`equals, getClass, hashCode, notify, notifyAll, toString, wait, wait, wait`

**Constructor Detail**

### RankedSetAnalysis

```
public RankedSetAnalysis(java.lang.String[] args)
```


**Method Detail**

### intersect

```
public void intersect()
```

---


### makeRankedHitsGraph

```
public void makeRankedHitsGraph()
```

---


### rankedIntersection

```
public static int[] rankedIntersection(GenomicRegion[] r)
```

:   Calculates the number of hits in the ranked matched list. As if one submitted a standard intersection
    analysis for every incremental rank.

---


### printRankedIntersection

```
public void printRankedIntersection(int[] hits)
```

:   Prints to screen the results of rankedIntersection(GenomicRegion[] r).

---


### containsMatchedRankIntersect

```
public static boolean containsMatchedRankIntersect(GenomicRegion r,
                                                   int testRank)
```

:   Checks to see if among the hits to a particular GenomicRegion they have an equal or smaller rank.

---


### fractionTotalIntersection

```
public double fractionTotalIntersection(GenomicRegion[] r)
```

:   Returns the total fraction of intersection.

---


### printRankedIntersectionAll

```
public void printRankedIntersectionAll(GenomicRegion[] r)
```

:   Rank analysis where each regions is check against the entire other list.

---


### intersectRegions

```
public boolean intersectRegions()
```

:   Loads regions with references to intersection partners.

---


### parseGenomicRegionsFile

```
public static GenomicRegion[] parseGenomicRegionsFile(java.io.File f)
```

:   Parses a regions file.

---


### main

```
public static void main(java.lang.String[] args)
```

---


### printDocs

```
public static void printDocs()
```

---


### processArgs

```
public void processArgs(java.lang.String[] args)
```

:   This method will process each argument and assign any new varibles

---


### getRegionsOne

```
public GenomicRegion[] getRegionsOne()
```

---


### getRegionsTwo

```
public GenomicRegion[] getRegionsTwo()
```

---


### getSetOneFile

```
public java.io.File getSetOneFile()
```

---


### getSetTwoFile

```
public java.io.File getSetTwoFile()
```

---


### getTextFrame

```
public TextFrame getTextFrame()
```

---


### isSavePngs

```
public boolean isSavePngs()
```

---


### getChartPanel

```
public org.jfree.chart.ChartPanel getChartPanel()
```

---


### setChartPanel

```
public void setChartPanel(org.jfree.chart.ChartPanel chartPanel)
```

---


### getChart

```
public org.jfree.chart.JFreeChart getChart()
```


---


|  |  |  |
| --- | --- | --- |
| **Overview** \| **Package** \| **Class** \| **Use** \| **Tree** \| **Deprecated** \| **Index** \| **Help** | |  |
| **PREV CLASS**   **NEXT CLASS** | **FRAMES**    **NO FRAMES**     **All Classes** |  |
| SUMMARY: NESTED \| FIELD \| CONSTR \| METHOD | DETAIL: FIELD \| CONSTR \| METHOD |  |


---
